# 1180. Comparing Changes in Pneumococcal Meningitis Incidence to all Invasive Pneumococcal Disease Following Introduction of PCV10 and PCV13: The PSERENADE Project

**DOI:** 10.1093/ofid/ofab466.1373

**Published:** 2021-12-04

**Authors:** Yangyupei Yang, Maria Deloria Knoll

**Affiliations:** Johns Hopkins Bloomberg School of Public Health, Baltimore, Maryland

## Abstract

**Background:**

The introduction of higher valency pneumococcal conjugate vaccines (PCV10 and PCV13) has reduced invasive pneumococcal disease (IPD) incidence. It is unknown whether the degree of reduction differs for pneumococcal meningitis, a small subset of pneumococcal disease but a major cause of severe childhood morbidity and mortality globally. We compared the impact of PCV10/13 on pneumococcal meningitis and all IPD by estimating the changes in incidence following the introduction of PCV10/13 among children < 5 years of age.

**Methods:**

Data on confirmed positive cases for pneumococcus in cerebrospinal fluid (CSF) were obtained directly from surveillance sites. PCV10/13 impact on all-serotype pneumococcal meningitis and all IPD were estimated using site-specific incidence rate ratios (IRRs) at each post-PCV10/13 year relative to the pre-PCV period, using Bayesian multi-level, mixed effects Poisson regression. All-site weighted average IRRs were estimated using linear mixed-effects regression. Results were stratified by product (PCV10 vs. PCV13) and amount of prior PCV7 use (none; some (1-3 years or 4-5 years with < 70% uptake); or many (≥ 4 years with ≥ 70% uptake).

**Results:**

40 surveillance sites (9 PCV10, 31 PCV13) in 28 countries, primarily high-income (82%) that had both CSF and IPD data were included in analyses. CSF+ accounted for 9.0% of IPD cases (IQR across sites: 6.2%-15.6%). The rate and amount of decline was generally similar between meningitis and IPD across all strata. At 5 years after PCV10/13 introduction, the IRRs across PCV7-use strata were 0.28-0.32 for pneumococcal meningitis and 0.22-0.43 for all IPD at PCV10-using sites, and 0.27-0.41 and 0.21-0.32, respectively, for PCV13-using sites. Only one site from the African meningitis belt contributed eligible data, which lacked pre-PCV data to estimate IRRs, but incidence rate of both IPD and meningitis decreased following PCV introduction.

Figure 1. All-Site Weighted Average Incidence Rate Ratios, Children < 5 years

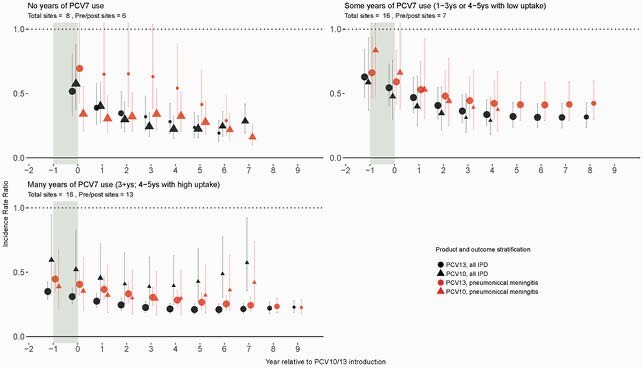

* Total sites indicate the number of sites with incidence rate data included and pre/post sites indicate the number of sites with both pre− and post−PCV data to estimate site−specific IRRs for each outcome. The size of point estimates is relative to the number of sites with both pre− and post− data. ** Year 0 indicates the year of PCV10/13 introduction and year −1 indicates the last year of PCV7 use prior to PCV10/13 introduction.

**Conclusion:**

Net declines in all-serotype IPD and CSF+ meningitis in children < 5 years were similar on average for both PCV10 and PCV13. Data from low-income, high-burden, and meningitis-belt regions were limited.

**Disclosures:**

**Maria Deloria Knoll, PhD**, **Merck** (Research Grant or Support)**Pfizer** (Research Grant or Support)

